# Isolation and biological characterization of tendon-derived stem cells from fetal bovine

**DOI:** 10.1007/s11626-016-0043-z

**Published:** 2016-04-29

**Authors:** Jinjuan Yang, Qianjun Zhao, Kunfu Wang, Hao Liu, Caiyun Ma, Hongmei Huang, Yingjie Liu

**Affiliations:** 1 Institute of Physical Education, University of Jimei, No. 185, Yinjiang Road, Jimei District, 361021 Xiamen City, Fujian Province People’s Republic of China; 2 Department of Animal Genetic Resources (AnGR), Institute of Animal Science, Chinese Academy of Agricultural Sciences, 100193 Beijing, People’s Republic of China

**Keywords:** Fetal bovine, Tendon-derived stem cells (TDSCs), Multi-differentiation, Markers, Genes, Immunofluorescence, RT-PCR, Therapeutic application

## Abstract

The lack of appropriate candidates of cell sources for cell transplantation has hampered efforts to develop therapies for tendon injuries, such as tendon rupture, tendonitis, and tendinopathy. Tendon-derived stem cells (TDSCs) are a type of stem cells which may be used in the treatment of tendon injuries. In this study, TDSCs were isolated from 5-mo-old Luxi Yellow fetal bovine and cultured in vitro and further analyzed for their biological characteristics using immunofluorescence and reverse transcription-polymerase chain reaction (RT-PCR) assays. It was found that primary TDSCs could be expanded for 42 passages in vitro maintaining proliferation. The expressions of stem cell marker nucleostemin and tenocyte-related markers, such as collagen I, collagen II, collagen III, and tenascin-C, were observed on different passage cells by immunofluorescence. The results from RT-PCR show that TDSCs were positive for collagen type I, CD44, tenascin-C, and collagen type III but negative for collagen type II. Meanwhile, TDSC passage 4 was successfully induced to differentiate into osteoblasts, adipocytes, and chondrocytes. Our results indicate that the fetal bovine TDSCs not only had strong self-renewal capacity but also possess the potential for multi-lineage differentiation. This study provides theoretical basis and experimental foundation for potential therapeutic application of the fetal bovine TDSCs in the treatment of tendon injuries.

## Introduction

Tendons, the critical structure of the musculoskeletal system, connect muscle to bone and transmit mechanical loading to provide joint function. Tendons are comprised of strong collagen fibrils and are constantly subjected to mechanical forces in vitro (Zhang and Wang 2010a). Tendons are frequently injured due to overuse or age-related degeneration during sports and rigorous physical activities, leading to long-term pain, tenderness, discomfort, dysfunction, and disability for athletes and individuals. Both acute tendon rupture and chronic tendinopathy regenerate and repair slowly and hardly regain the integrity of strength of a normal undamaged tendon. The natural repair process is not sufficient to achieve complete functional restoration. The pathogenesis is unclear and its treatment is primarily symptomatic and conservative. Surgical suture of the broken tendon or surgical excision of the pathological tissue was frequently followed by severe postoperative complications. The efficacy of current treatments is poor causing a huge burden on health care systems (Sharma and Maffulli 2006), and the treatment of tendon injuries remains a challenge to both general medicine and sports medicine. Improvement of the current the therapeutic strategies is urgently needed. Stem cell therapy has recently received increasing attention as a potential new strategy for the treatment of tendon injuries.

Stem/progenitor cells are unspecialized cells that can self-renew and have the ability to differentiate into cells with highly specialized functions. Stem cells plays important role in the process of growth and development for individuals. However, increasing evidence suggests that they may also have great effects on pathological conditions (Caplan and Bruder 2001). Currently, stem cell research mainly focuses on the isolation, culture, identification and characterization, and proliferation in vitro (Zhang and Wang 2010a). Stem cells have been isolated from different tissues of various species, including human, rabbit, rat, mouse, and horse and used to regenerate functional tissues and organs (Bi et al. 2007; Rui *et al.*^2010^, ^2013^; Lovati et al. 2011; Zhang and Wang 2010b). Mesenchymal stem cells (MSCs), being multi-potent and having the ability to self-renewal, can differentiate into lineages of mesenchymal tissues including bone, cartilage, fat, and tendon (Tan *et al.*^2012^). Different sources of MSCs have been studied for their refine effect on tendon repair, but ectopic bone and tumor formation have been reported in some special circumstances after their transplantation of stem cells (Harris *et al.*^2004^). Other studies have also shown that multi-potent stem cells indwell in tendon and ligament tissues. Compared with other stem cells, tendon stem/progenitor cells, also called tendon-derived stem cells (TDSCs), possess both self-renewal and multi-lineage differentiation capacity and are particularly clinically relevant for tendon repair duo to the origin of tissue from which they are isolated (Fenwick *et al.*^2002^; Salingcarnboriboon *et al.*^2003^). TDSCs may be a better choice for the study of the potential roles of stem cells in tendon pathology and pathological mechanism of tendinopathy. In this study, we isolated TDSCs from fetal bovine Achilles’s tendon, observed multi-lineage differentiation potential, and identified their biological characteristics. The results indicate that tendon progenitor cells isolated from fetal bovine possess properties of stem cells and may be ideal candidates for cell-based therapy in treatment of tendon injuries.

## Materials and Methods

### **Experimental animal**

Animal experiments were performed in accordance with the guidelines established by the Institutional Animal Care and Use Committee of the Chinese Academy of Agriculture of Sciences. The Chinese Luxi Yellow cattle used in this study was provided by the Chinese Academy of Agriculture Sciences’ Farm. Fetuses (4∼5 mo old) were removed by caesarean section, and a total of 10 fetuses were used in this research. Fetus samples were collected, maintained in an ice tray, and immediately transported to the laboratory within 4 h after the caesarean section.

### **Isolation and culture of TDSCs**

Achilles tendon tissues were excised from both lower limbs of each fetal bovine and dissected with following procedures: the peritendinous connective tissues of tendon were stripped off and washed with sterile phosphate-buffered saline (PBS) for at least six times. The trimmed tissues were cut into 1-mm^3^ pieces using ophthalmic scissors. The comminuted tissues were digested with 0.1% collagenase I (3 mg/ml; Sigma, St. Louis, MO) for 1 h at 37°C. Postdigestive tendon tissues were passed through a 70-μm mesh filter to yield single-cell suspension after lower glucose-DMEM (L-DMEM; Gibco, Carslbad, CA) containing 15% FBS was added to terminate the reaction. The cell suspension was centrifuged at 1200 r/min for 10 min and the supernatant was discarded. The cells were resuspended in L-DMEM complete culture medium supplemented with 15% FBS (Invitrogen, Carlsbad, CA), 100 U/ml penicillin (Invitrogen), 100 mg/ml streptomycin (Invitrogen), 2.5 ng/ml bFGF (Peprotech, Rocky Hill, TX), and 2 mM L-glutamine (Invitrogen). The cell suspension was seeded in 60-mm petri dish (Wuxi Nest Biotechnology Co. Ltd., Jiangsu, China) at 1 × 100 cells/ml and incubated at 37°C with 5% CO_2_ (Lui 2015). After 36 h of seeding, the cells were washed twice with L-DMEM media to remove non-adherent cells. When adherent cells reached about 70–80% confluence, the adherent cells were digested with 0.125% and trypsin 0.02% EDTA and subcultured into new petri dishes at a ratio of 1:1. Normally, after three or four passages (*P*), freshly isolated cells were homogenous and purified.

### **Cell population growth dynamics**

To assess growth dynamics of TDSCs, cells of P4, P15, P25, and P35 were seeded in 24-well plates at a density of 1 × 10^4^ cells/well per passage and continually cultured for 8 d. Cells were counted by blood counting instrument (3 wells per time, and the mean value of the cell counting was calculated) (Bai *et al.*^2011^). The growth curves of different passages were plotted by the mean values of population doubling time (PDT) using the formula PDT = (*t* − *t*_0_) lg2/(lg*N*_*t*_ − lg), where *t*_0_ is the starting time of culture, *t* is the termination time of culture, *N*_0_ is the initial cell number of culture, and *N*_*t*_ is the ultimate cell number of culture.

### **Colony-forming cell assay**

The P4, P15, P25, and P25 cells were seeded in 6-well plates at 1 × 10^4^ cell/well and cultured for 8 d. The numbers of colony-forming units (CFU) were counted to calculate colony-forming rate, which is formulated as colony-forming unit cell number/starting cell number per 6 well × 100%. This procedure was repeated six times for each passage.

### **Karyotype analysis**

Karyotype of P10 cells was analyzed as previously described (Baran and Ware 2007). Cells were harvested at 80∼90% confluence, subjected to hypotonic treatment, and fixed with the combination of glacial acetic acid and acetic acid. The chromosome numbers were counted from 100 spreads under an oil immersion objective upon Giemsa staining. Relative length, arm ratio, and centromeric index were calculated according to a protocol reported by Kawarai *et al.* (2006).

## Identification of TDSCs

### **Immunofluorescent detection of cell surface markers**

To identify the expression of markers in the isolated cells, immunofluorescence histochemical staining was performed as described previously for the detection of collagen type I, collagen II, collagen type III, CD44, and tenascin-C (Ni *et al.*^2012^). In brief, cells at P4, P15, P25, and P35 were seeded in 6-well plates, respectively, and cultured in complete cell culture medium. After culturing for 2 d, cells were washed three times with PBS, then fixed with 4% paraformaldehyde at room temperature for 30 min, and were washed with PBS three times. In order to permeabilize cytolemma, cells were incubated with 0.25% Triton X-100 (Sigma-Aldrich, St. Louis, MO) for 20 min and were washed again with PBS three times, then cells were incubated with 10% BSA for 1 h at room temperature, and received primary antibodies (rabbit anti-collagen I antibody, rabbit anti-collagen II antibody, rabbit anti-collagen III antibody, rabbit anti-CD44 antibody, and rabbit anti-tenascin-C antibody (all from Bioss, Woburn, MA)) of target cell markers and incubated at 4°C overnight. The primary antibody was removed and cells were washed three times with PBS. Cells were incubated with the secondary antibodies in 1% PBS for 1 h at room temperature; FITC-goat anti-mouse secondary antibody (1:100, Zhongshan Golden Bridge, Beijing, China) was added and incubated at room temperature in a darkroom for 1 h. The secondary antibody solution was decanted and washed three times with PBS in dark. Eventually, the cells were incubated in 1 μg/ml DAPI (Sigma-Aldrich, St. Louis, MO) for 15 min and washed three times with PBS. Finally, photomicrographs were taken using a Nikon TE-2000-E confocal microscope with an attached Nikon ZE-1-C1 3.70 digital camera system (Nikon, Tokyo, Japan).

### **Reverse transcription-polymerase chain reaction of TDSCs**

To characterize the expression of related genes by reverse transcription-polymerase chain reaction (RT-PCR), total cellular RNA was obtained as described previously (Zhang and Wang 2010a). Isolated cells of P4, P15, P25, and P35 were collected and extracted by using TRIzol reagent (Invitrogen, Carlsbad, CA). The RNA concentrations of different passage were measured by absorbance at 260 nm with a spectrophotometer. RNAs were transcribed followed by 35 PCR cycles using RNA PCR Kit version 3.0 (TaKaRa, Dalian, China). RT-PCR was performed in a 20 μl mixture containing 10-μl 5× PCR buffer, 7 μl ddH_2_O, 1-μl forward primers, 1-μl reverse primers, and 1 μl cDNA. The cycling conditions consisted of 1 cycle of initial 2 min at 94°C, then followed by 30 cycles of 30 s at 94°C (for denaturation), 30 s at 50∼60°C (for annealing), and 2 min at 72°C (for extension). PCR products were detected by 2.0% agarose gel electrophoresis. In this experiment, specific primers were used for the following genes: collagen I, tenascin-C, and collagen III (tenocyte-related gene); CD44 (specific surface markers of MSCs); lipoprotein lipase (LPL) gene and peroxisome proliferator-activated receptor-γ (PPAR-γ gene; two adipocyte-related genes) (Ruge *et al.*^2004^); aggrecan (ACAN), collagen II, and Sox9 (chondrocyte-related gene) (Bell *et al.*^1997^; Kanai and Koopman 1999); and runt-related transcription factor 2 (Runx2), alkaline phosphatase (ALP), and osteopontin (OPN; osteocyte-related gene). Glyceraldehyde-3-phosphate dehydrogenase (GAPDH) was used as an internal control. All primers were designed by Primer Premier 5.0 software, and designed primers were synthesized by Sangon Biotech Co., Ltd. (Shanghai, China). Table 1 shows the related information.

**Table 1:** The primer sequences for RT-PCR

Gene names	Primer sequences	Tm (°C)	Product length (bp)
Collagen I	F 5′-CAAGAAGAAGACATCCCACCA-3′	58	369
	R 5′-GGACTTTGGCGTTAGGACAG-3′		
Collagen II	F 5′-GGATGCCATGAAGGTTTTCTGC-3′	62	424
	R 5′-CATAGTCTTGCCCCACTTACCG-3′		
Collagen III	F 5′-CTCCCATCTCGGTCAGTCTT-3′	60	184
	R 5′-TTCTCCAAACGGGATTTCAG-3′		
Tenascin-C	F 5′-CCTGGAATCTCCACGGAGTG-3	59.8	298
	R 5′-GAACCTCACAGTGACCAGGG-3		
CD44	F 5′-TGCTGCGAACAACACG-3′	53	268
	R 5′-GCCGCTGCTCATCTCA-3′		
GAPDH	F 5′-TGCCAACATCAAGTGGGGTG-3′	62	415
	R 5′-GTCCCTCCACGATGCCAAAG-3′		
LPL	F 5′-ATGAACTGGATGGCGGATGA-3′	62	308
	R 5′-GGTTGGAAAGTGCCTCCGTTA-3′		
PPARr	F 5′-GTTGACACAGAGATGCCGTTTT-3′	58.2	360
	R 5′-ATAGTGGAACCCTGACGCTTT-3′		
Sox9	F 5′-GTGCTCAAGGGCTACGACTGG-3′	62	362
	R 5′-CGTTCTTCACCGACTTCCTC-3′		
OPN	F 5′-CGATGATGATAACAGCCAGGAC-3′	59.6	157
	R 5′-AAACGGAGTGAAAACTGCGATT-3′		
Runx2	F 5′-GGCGTTCTGTGGTTTCATACT-3′	62	301
	R 5′-AACTTGCCTGCTCCTTCAGAT-3′		
ACAN	F 5′-TCGTGGTAAAACCGACTTCCTC-3′	60.1	194
	R 5′-GGCGAGATGTGCGTCTGTTC-3′		
ALP	F 5′-TCCACGAGCAGAACTACATCC-3′	59.3	304
	R 5′-AGGCAGACTTTGGTTTCCTG-3′		

## Multi-Potent Differentiation of TDSCs In Vitro

### **Adipogenic differentiation of TDSCs**

To test adipogenic potential, cells in control group were cultured in complete culture medium, while the induced group cells were cultured in adipogenic induction medium which consisted of L-DMEM added with dexamethasone (1 mM), insulin (10 mg/ml), indomethacin (100 mM), and isobutylmethylxanthine (IBMX; 0.5 mM) in 6-well plates at a density of 2 × 10^5^ cells/well. After 18-d culture, TDSCs cultured in complete medium and in adipogenic induction medium were stained using 0.3% Oil Red O (Sigma) for adipogenesis; the procedures are displayed as follows: removal of culture medium, then washing of the cells with PBS three times, fixing the cells with 4% paraformaldehyde for 30 min at room temperature, and washing the cells with PBS again; finally, cells were incubated with Oil Red O solution for 30 min and then washed with PBS three times. Stained samples were examined on an inverted microscope (Gimble *et al.*^1995^).

### **Osteogenic differentiation of TDSCs**

As a test of osteogenic potential of TDSCs, cells were plated at 6 × 10^2^ cells/cm^2^ in 6-well plate, respectively, in complete culture medium and in osteogenic induction medium, which consisted of basic growth medium supplemented with 0.5 μm dexamethasone, 50 mM ascorbic acid, and 10 mM β-glycerophosphate (all from Sigma). After 4-wk induction, cells cultured in complete culture medium and in osteogenic induction medium were stained using Alizarin Red S for osteogenesis. The following procedures include fixing the cells with 4% paraformaldehyde for 30 min at room temperature, rinsing with PBS three times, staining with Safranin O (Sigma, St. Louis, MO) for 30 min, and then washing with PBS three times. Stained samples were examined on an inverted microscope (Bi *et al.*^2005^).

### **Chondrogenic differentiation of TDSCs**

As a test of chondrogenic potential of TDSCs, cells were cultured in 6-well plates, respectively, in complete culture medium and in osteogenic inducing medium, which consisted of L-DMEM added with proline (40 mg/ml), dexamethasone (39 ng/ml), TGF-β_3_ (10 ng/ml), ascorbate 2-phosphate (50 mg/ml), sodium pyruvate (100 mg/ml), and insulin-transferrin-selenious acid mix (50 mg/ml). After 26-d culture, the cells cultured in two kinds of culture medium above were stained with Safranin O dye, fixed with ice cold ethanol for 1 h, then washed with PBS three times, stained with Safranin O for 30 min at room temperature, and then washed with PBS or distilled water three times. The stained samples were examined on an inverted microscope (Wang *et al.*^2003^).

## Results

### **Isolation, culture, and morphology of TDSCs**

**Figure 1.**
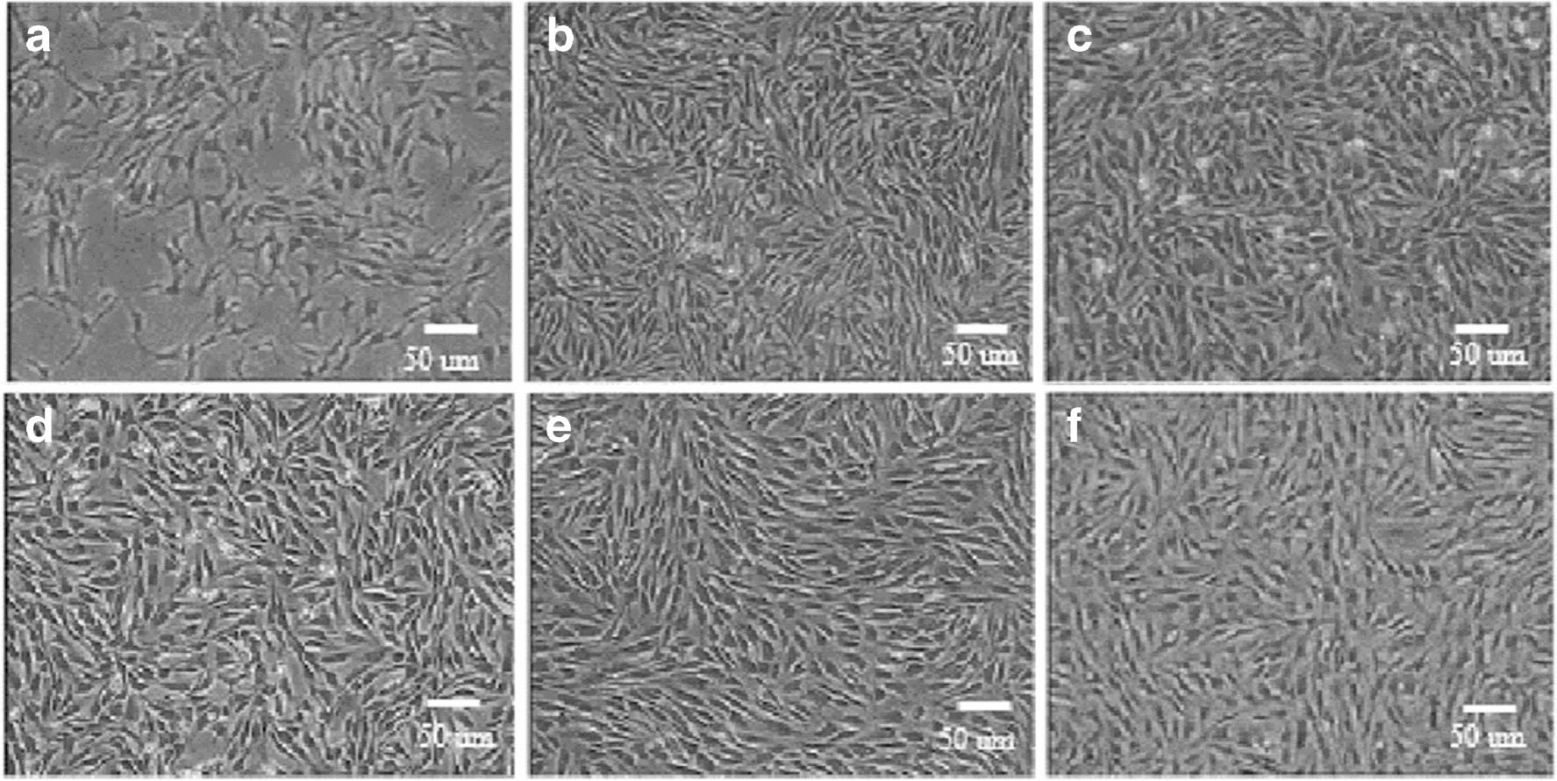
Cell morphology of primary cultured and subcultured. (*a*) Primary cells after culture for 48 h, many cells began to adhere and stretch. (*b*) Passage 4 adherent cells were spindle-shaped or fusoid; cell morphologies at passage 15 (*c*), passage 25 (*d*), passage 35 (*e*), and passage 42 (*f*). Cells of passage 42 (*f*) displayed representative senescent appearances (*scale bar* = 50 μm).

Primary cells isolated from Achilles tendon tissues began to attach to the culture petri dishes after 48 h (Fig. 1*a*). Approximately 5 d later, cells reached about 70∼80% confluence and exhibited fusiform morphology. Cells at passage 4 were used in the following experiments (Fig. 1*b*). There were no obvious morphological differences among different passages, and the morphology remains stable after several passages (Figs. 1*c*, *d*, *e*). In this study, cells isolated from fetal bovine Achilles tendons were subcultured up to passage 42 with most cells displaying a senescent appearance (Fig. 1*f*), indicating that cells isolated from fetal bovine were able to proliferate for a long time in vitro.

### **Growth dynamics of TDSCs**

**Figure 2.**
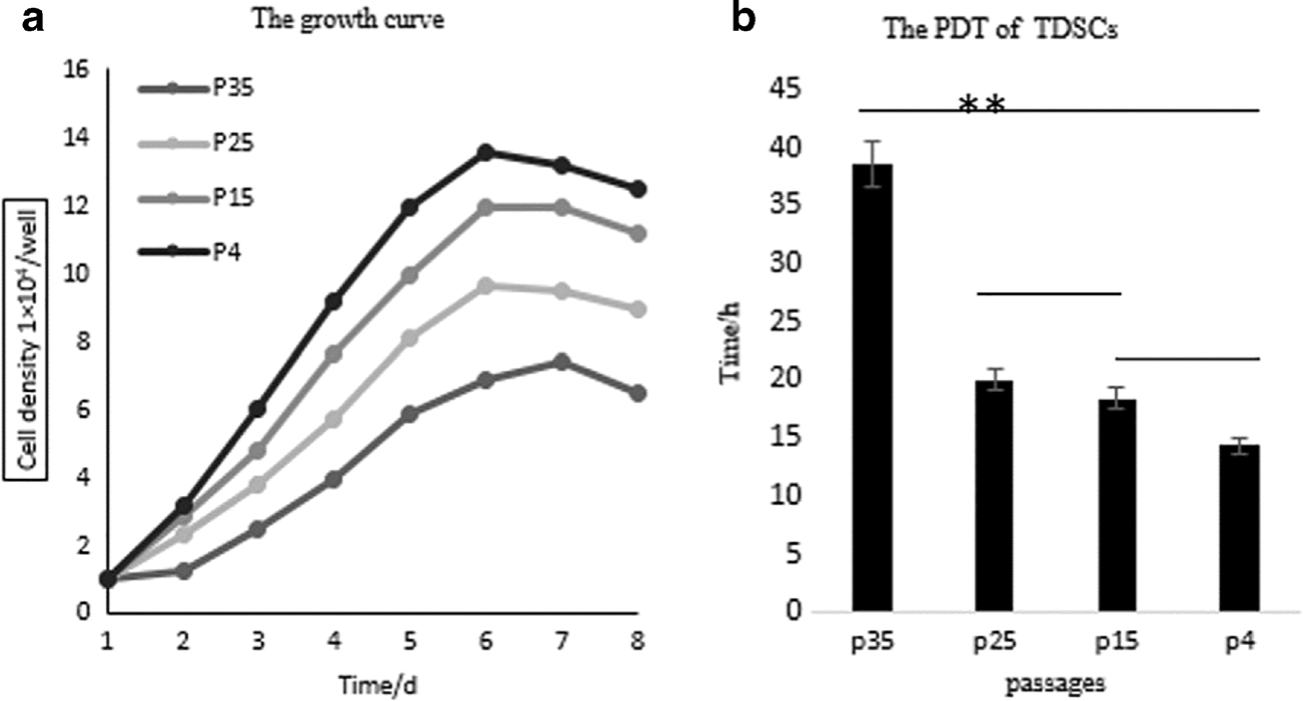
Growth curves and PDT of the TDSCs. The growth curves of P4, P15, P25, and P35 cells were typically sigmoidal, consisting of a latent phase, a logarithmic phase, and a plateau phase. The population doubling time (PDT) was plotted in Fig. 2 separately. *Lines on the top of those bars* show comparison of the PDT at different passages by *t* test, where **P < 0.01 represents the PDT of P35 cells versus that of P25, P15, and P4, respectively, while comparisons between P25 and P15, P15 and P4, and P4 and P25 represent a positive Ho hypothesis.

The growth dynamics of cells from P4, P15, P25, and P35 were shown as the growth curves, which were all typically sigmoidal. Cells at these different passages entered logarithmic phase after about 4 d, followed by plateau phase after 7 d, and began to decline quickly (Fig. 2). The average population doubling times of cells of these four passages were 14.32, 18.29, 19.69, and 38.49 h, respectively, which were shown by bar chart (Fig. 2*b*). The PDT of cells at passages 4, 15, 25, and 35 showed a rising trend, among which the PDT of passage 35 cells was significantly longer than others (*P* < 0.01), while passage 4, passage 15, and passage 25 cells showed no significant differences (*P* > 0.01).

### **Colony-forming cell assay**

**Figure 3.**
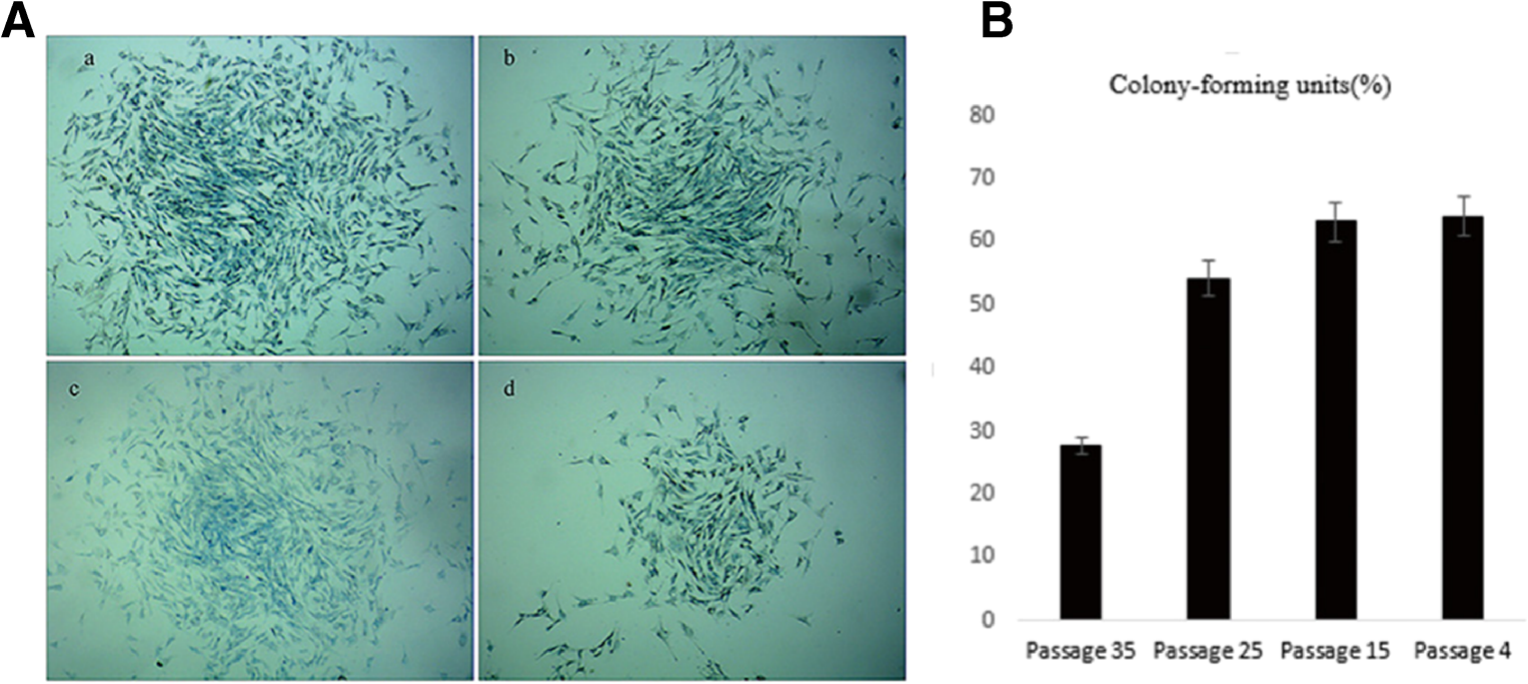
Colony-forming cell assay. (*A*) Colony-forming units of P4 (*a*), P15 (*b*), P25 (*c*), and P35 (*d*) tendon-derived progenitor cells. (*B*) Plot of the colony-forming rate counting. The colony-forming rates are higher in P4, P15, P25, and P35 than that of P38, but colony-forming rates did not disappeared with increasing passage number (*scale bar* = 50 μm). *Lines on the top of those bars* show comparison of the average of cell colony-forming rates at different generations by *t* test, among which, **P < 0.01 represents a negative Ho hypothesis for colony-forming rates for P35 versus P25, P15, and P4, respectively, while comparisons between P25 and P15, P15 and P4, and P4 and P25 represent a positive Ho hypothesis.

Colony-forming of different passages of the TDSCs were observed under the microscope after 4 d (Fig. 3*A*). The colony-forming rates for passages 4, 15, 25, and 35 were 64, 63.1, 54.19, and 27.81%, respectively (Fig. 3), which indicated the self-renewal and proliferation ability of the different passage of fetal bovine TDSCs cultured in vitro.

### **Karyotype analysis**

**Figure 4.**
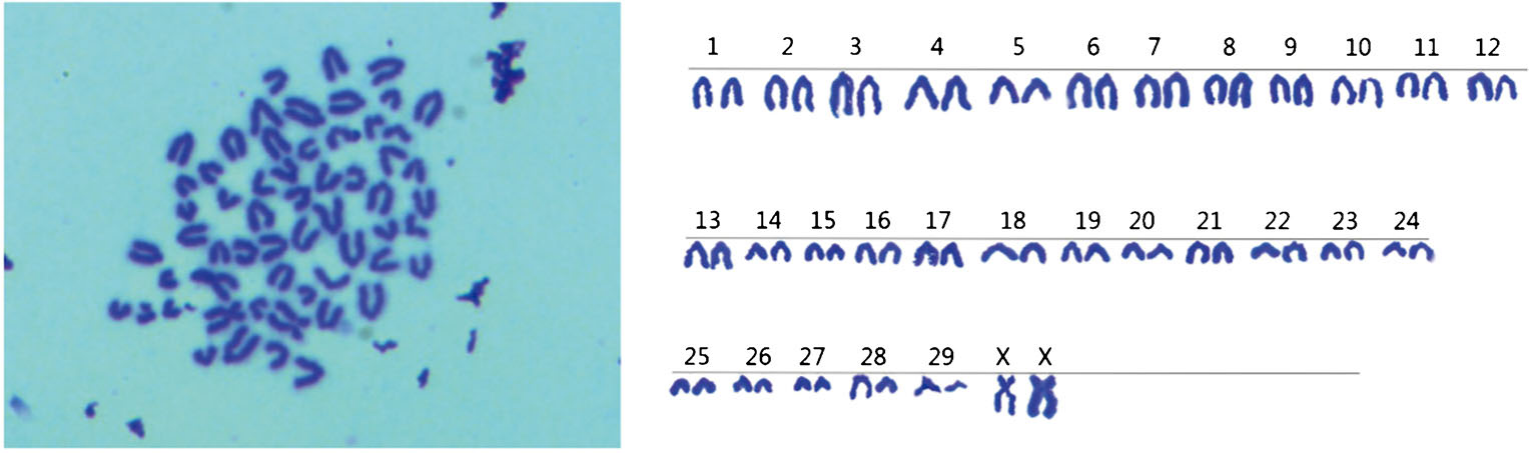
Karyotype of fetal bovine TDSCs XX (♀) type. The diploid chromosome number of fetal bovine TDSCs was 2*n* = 60, consisting of 29 pairs of autosome and 1 pair of sex chromosome. A representative XX (♀) was shown (*n* = 60).

The diploid chromosome number of fetal bovine derived of TDSCs was 2*n* = 60, consisting of 1 pair of sex chromosome and 29 pairs of autosome, with the sex chromosome type that was XX(♀)/XY(♂). In this study, the chromosome numbers were counted in 100 spreads in different passages. The results showed that diploid cells were 97%, indicating that cells cultured in vitro possessed of genetic stability. The karyotype of TDSCs is shown in Fig. 4.

### **Immunofluorescence of the TDSCs**

**Figure 5.**
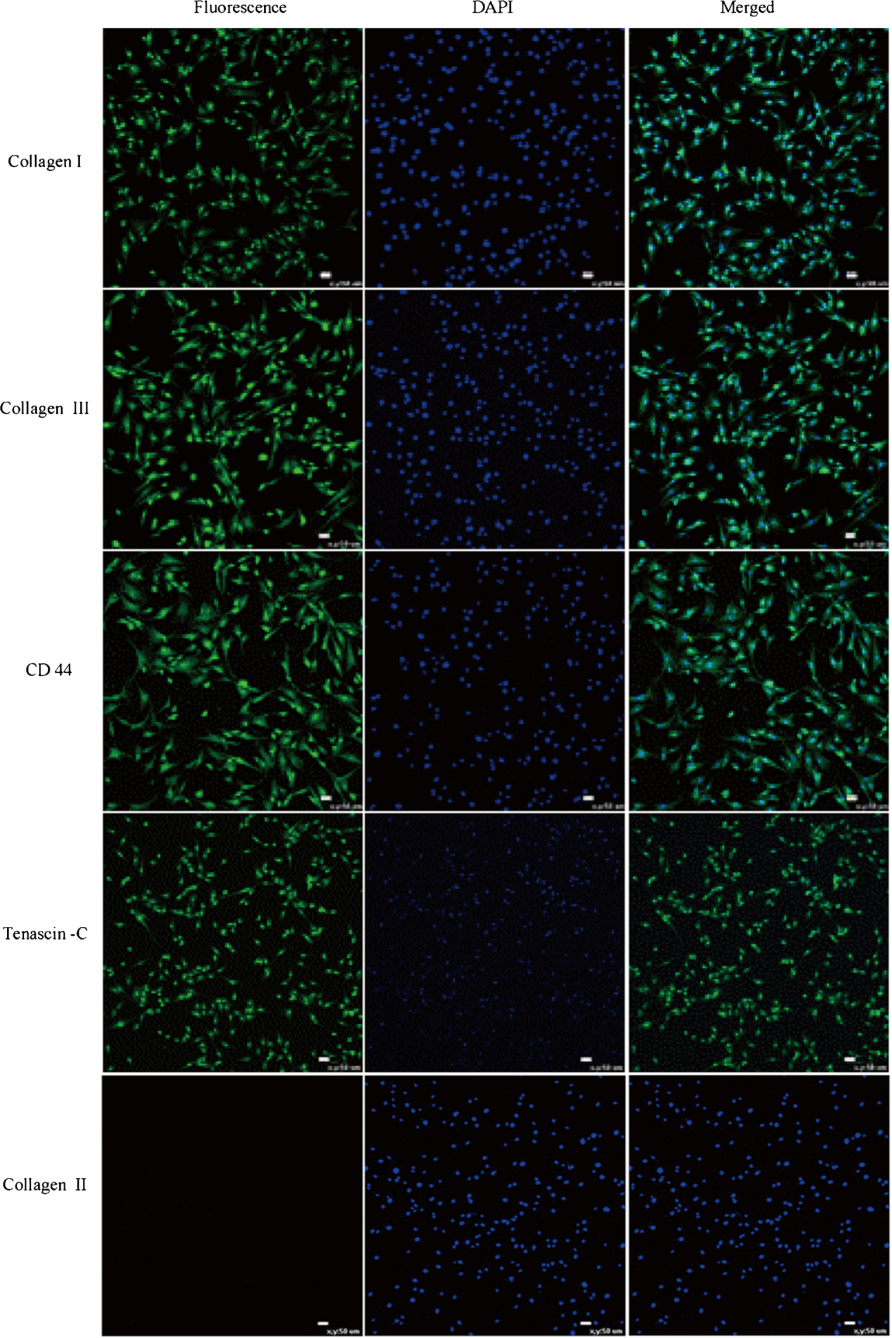
Characterization of fetal bovine derived TDSC surface markers by immunocytochemistry. Immunofluorescence staining indicating that collagen I, collagen III, CD44, and tenascin-C are positively expressed, while collagen II was negative. Fluorescent markers were shown in the *left panels* (*green*) and nuclei stained with DAPI in the *middle panels*. Merged images in the *right panels* (*scale bar* = 50 μm).

The specific surface antigen markers of TDSCs were detected via immunofluorescence and RT-PCR assays. The results of immunofluorescence staining showed that the TDSCs of different passages were collagen I, collagen III, CD44, and tenascin-C positive and collagen II negative (Fig. 5).

### **RT-PCR analysis of TDSCs**

**Figure 6.**
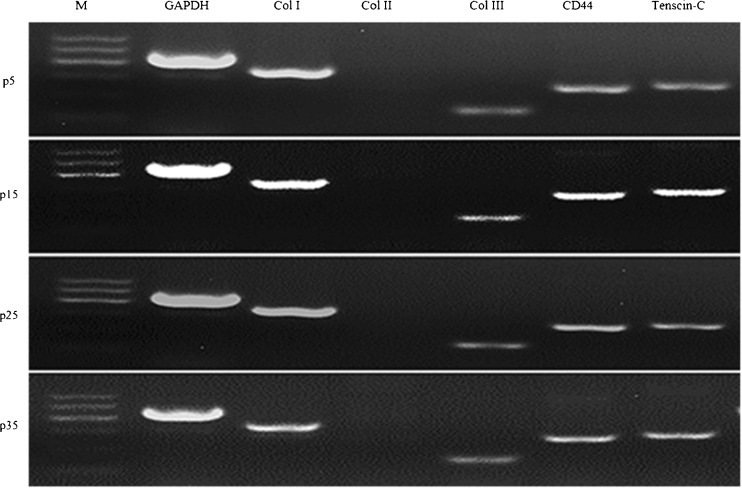
Detection of collagen I, collagen II, and collagen III by RT-PCR analysis. Results show that TDSCs at passage 4, passage 15, passage 25, and passage 35 were positive for collagen I, CD44, tenascin-C, and collagen III and negative for collagen II. *M* marker 1; GAPDH served as the internal control.

RT-PCR experiments indicated that the TDSCs at different passages expressed tendon specific genes collagen I, tenascin-C, and collagen III, and also expressed CD44 that is the specific gene of MSCs, but did not express collagen II gene (Fig. 6).

### **Multi-potent differentiation of TDSCs in vitro**

We tested multi-differentiation potential of fetal bovine TDSCs toward osteogenesis, adipogenesis, and chondrogenesis in vitro as described previously. Cells were successfully induced to differentiate into osteoblasts, adipocytes, and chondrocytes in induced medium, respectively.

### **Adipogenic differentiation of TDSCs**

**Figure 7.**
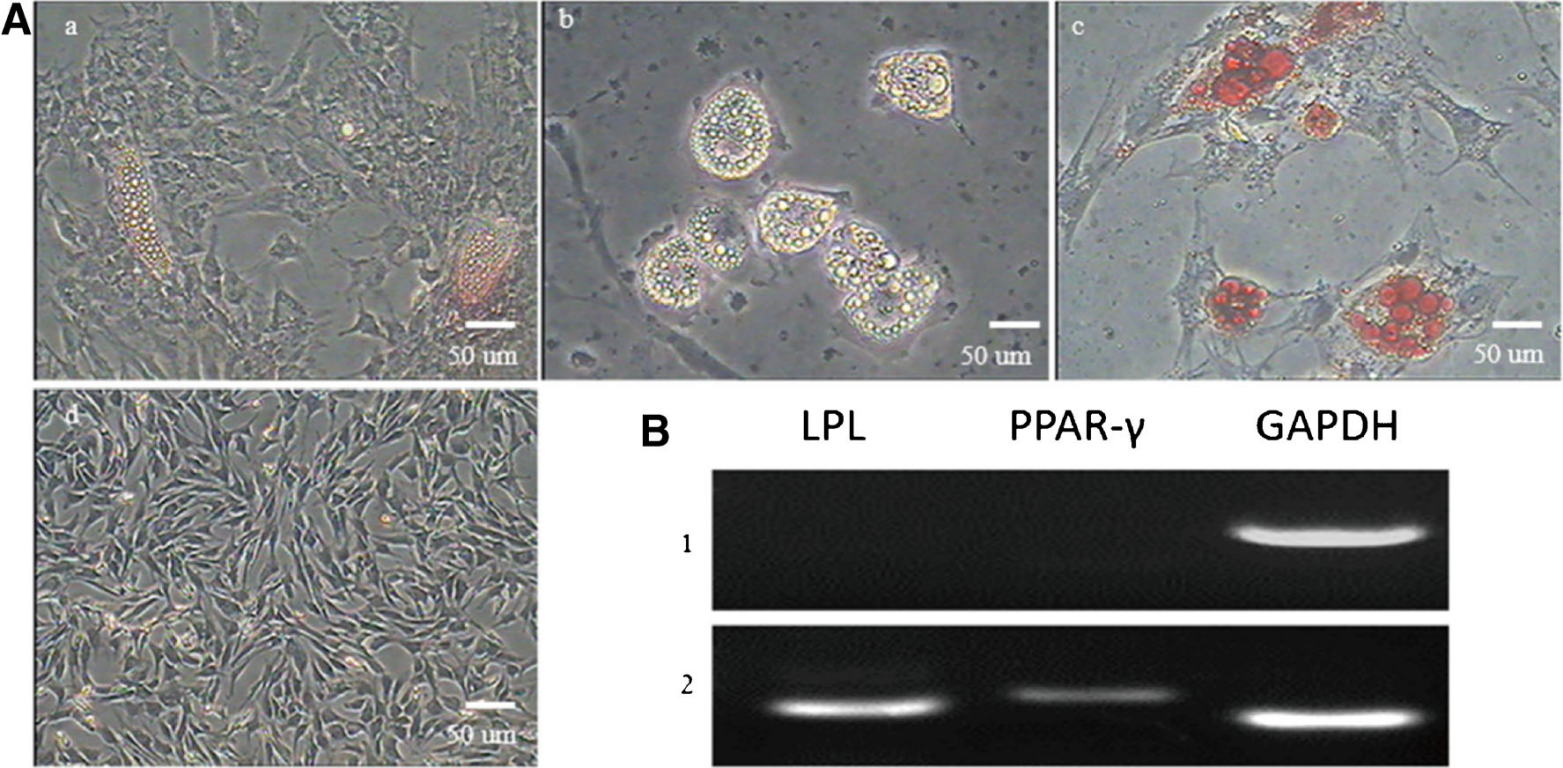
Adipogenic differentiation of TDSCs. (*A*) (*a*) After inducting for 11 d, TDSCs were oblate shape with some intracellular lipid droplet. (*b*) Droplets increased and aggregated to form larger ones as induction progressed. (*c*) Oil Red O staining for lipid droplets. (*d*) Negative controls. Cells cultured in complete medium did not alter morphology and were negative by Oil Red O staining. (*B*) Expression of adipocyte-specific genes, including PPAR-γ and LPL, were detected by RT-PCR. These genes were not expressed in the control group, (1) control group and (2) induced group (*scale bar* = 50 μm).

Adipogenic differentiation of TDSCs was demonstrated by Oil Red O staining (Fig. 7*A*). After incubating in adipogenic induction medium for 11 d, TDSCs changed from a shuttle shape to an oblate shape and contained many intracellular lipid droplets that increased and aggregated to form a larger droplet at day 21 postinduction. After inducting for 21 d, RT-PCR results showed that the cells expressed adipocyte-specific genes PPAR-γ and LPL, whereas these genes were not expressed in the control group (Fig. 7*B*).

### **Osteogenic differentiation of TDSCs**

**Figure 8.**
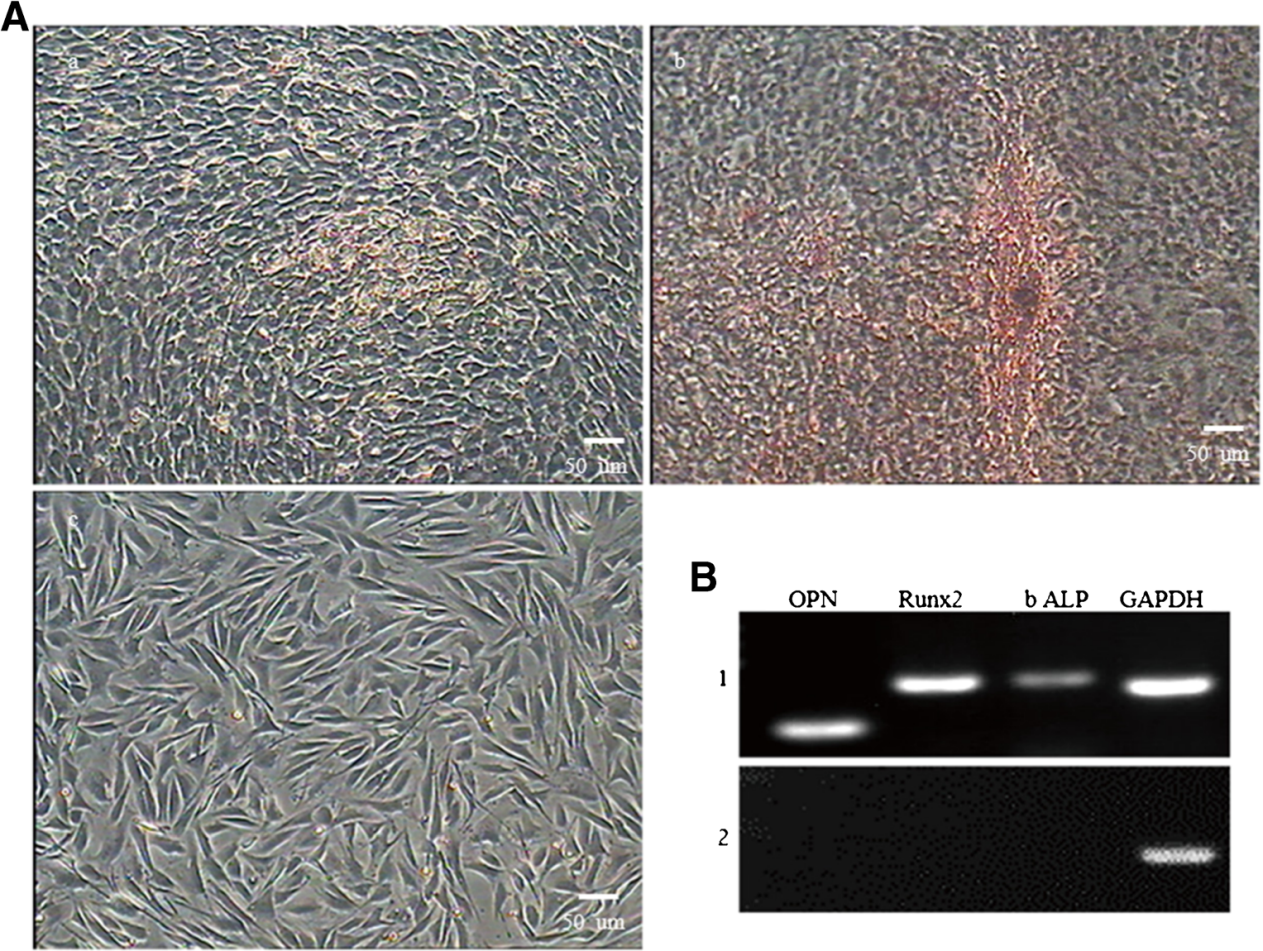
(*A*) Osteogenic differentiation of TDSCs. (*a*) The cells changed from long fusiform to triangle in shape and formed some calcified nodules after induction for 15 d. (*b*) Calcified nodules increased in number and became larger after induction for 28 d, and they were positive for Alizarin Red staining. (*c*) The control group. (*B*) RT-PCR revealed the expression of osteoblast-specific genes of Runx2, bLAP, and OPN in the induced group, whereas these genes were not expressed in the control group. (1) Runx2, bLAP, and OPN were positive in the control group. (2) Runx2, bLAP, and OPN were negative in the inducted group. GAPDH served as the internal control (*scale bar* = 50 μm).

After incubation in osteogenic medium for 15 d, TDSCs showed obvious morphological changes. After 28 d of induction, the cells became aggregated and formed mineralized nodules that were stained with Alizarin Red (Fig. 8*A*). In addition, the number and size of nodules were increased as induction progressed, whereas cells cultured in complete medium showed no morphological changes and were negative for Alizarin Red staining. Osteogenic differentiation of TDSCs was also analyzed by RT-PCR. Osteogenic-specific genes OPN, bALP, and Runx2 were expressed in the induced group but were not express in the control group (Fig. 8*B*).

### **Chondrogenic differentiation of TDSCs**

**Figure 9.**
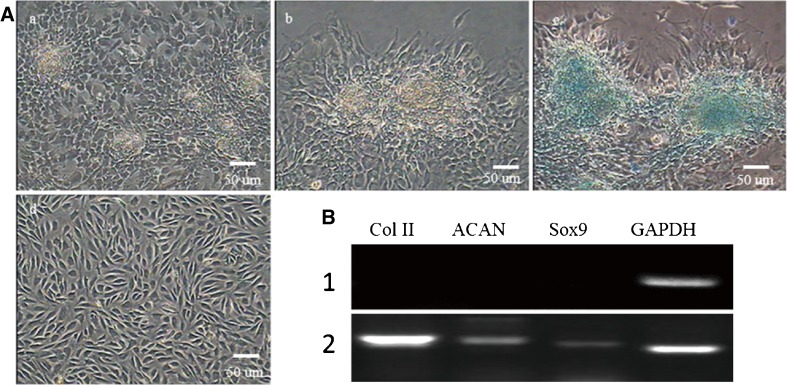
(*A*) Chondrogenic differentiation of TDSCs. (*a*) After induction in chondrogenic medium for 7 d, the induced cells changed from long fusiform to elliptic in shape and formed calcified nodules. (*b*) Calcified nodules were bigger in chondrogenic medium after 15 d. (*c*) Induced cells were positive for Alcian blue staining. (*d*) Control cells. Cells cultured in complete medium showed no morphological change and were negative for Alcian blue staining. (*B*) After inducting for 2 wk, RT-PCR revealed the expressions of chondrogenic-specific genes collagen II, Sox9, and ACAN. (1) Collagen II, Sox9, and ACAN were negative in the control group. (2) Collagen II, Sox9, and ACAN were positive in the inducted group; GAPDH served as the internal control (*scale bar* = 50 μm).

After incubation in chondrogenic differentiation medium for 7 d, TDSCs were about 90% confluent and still remained TDSC morphology. After 15 d of induction, the cells were densely packed, with phase-bright cell clusters. These clusters formed 3D nodules with cells clustered into “rosette-like” morphology (Fig. 9*A*). During the third week of induction, the flatter cells surrounding the 3D cell aggregates began to detach, leaving behind the 3D cell aggregates, and were stained with Alcian blue. Past 3 wk postinduction, the expression of specific genes, including collagen II, ACAN, and Sox9, were analyzed by RT-PCR (Fig. 9*B*).

## Discussion

Tendons are composed mostly of parallel arrays of collagen fibers, which transmit muscular forces to bone and are susceptible to pathological changes due to overuse, age-related degeneration, or rigorous physical activities. The process of natural healing usually results in the formation of fibrotic scar tissue, which has poor tissue quality and the inferior mechanical properties (Butler *et al.*^2004^). Currently, the treatment of tendon injuries is usually symptomatic. Stem cell therapies, based on their self-renewal capacity and regenerative ability, have been studied for the treatment of tendon injuries, but a better understanding of the biological characteristics of stem cells in tendon may provide an insight of the pathological mechanisms of tendon injuries. Therefore, there is great significance to study the biological characteristics of tendon stem cells in vitro.

Studies have shown that stem cells increase in number and decrease in proliferative capacity with increasing age (Lui and Chan 2011). This study chose 4–5 mo old fetal bovine to isolate TDSCs; our samples were offered by the Chinese Academy of Agriculture Sciences’ farm which is near from our lab, and the purity of Luxi Yellow cattle was guaranteed. Our results showed that TDSCs from Achilles tendon mid-substance of fetal bovine were successfully isolated and were subcultured in the completed medium up to passage 42, indicating that stem cells isolated from Achilles tendon can proliferate for a long time. Their self-renewal ability was assessed by cell population growth dynamics and colony-forming cell assay.

By using of immunofluorescence and PT-PCR methods, we identified that a number of specific markers including collagen I, collagen II, collagen III, tenascin-C, and CD44 of the fetal bovine TDSCs were tested. Collagen I and collagen III are important structural protein of the extracellular matrix in tendon. Collagen II is secreted by chondrocytes and promotes the healing of fractures and the relief of arthroses. CD44 is a cell surface glycoprotein involved in cell-cell interaction, migration, and adhesion, which participates in various cellular function including lymphocyte activation, recirculation, and homing. Tenascin-C, also known as cell integrin C, is a type of extracellular matrix which participates in the regulation of cell morphology. The results show that cells isolated from fetal bovine Achilles tendon tissue were positive for collagen I, collagen III, CD44, and tenascin-C and were negative for collagen II.

Multiple differentiation potential is the most remarkable characteristic of stem cells. Cultured in different induction medium, stem cells were able to differentiate into various cell lineages. The development and function of tissue stem cells are related to transcription factors and extracellular signals in vivo. In this study, TDSCs originate from the mesoderm but can be differentiated into cells that could be of a mesodermal or ectodermal origin, such as chondrocytes, osteocytes, and adipocytes. Relevant gene expressions of these cell types were detected by different cell staining reagent and RT-PCR, which demonstrated that different induction factors affect the differentiation of TDSCs and that TDSCs from fetal bovine can be induced into different cells under different induction conditions. Although its multi-lineage differentiation was successful in vitro, the mechanisms of differentiation remain unknown, and there still remains some drawbacks in the use of stem cells in tissue regeneration in vitro, such as the low-induction efficiency and the cell purity in the process of cell cultivation.

## Conclusions

This study established an optimal method for isolation and culture of TDSCs in vitro and investigated their proliferation and self-renewing ability by cell growth curve and clonal efficiency, then identified their specific marker expression by immunofluorescence staining and specific gene expression by RT-PCR. TDSCs were also induced to adipocytes, chondrocytes, and osteocytes with different induction medium, providing strong evidence for the multi-potential of TDSCs in vitro. These results demonstrated that fetal bovine TDSCs not only have strong self-renewal ability but also possess the potential to differentiate into different types of cells. These results may lay a foundation for fetal bovine TDSCs as a model of tendon pathological mechanism and repairman mechanism study.
